# 

**Published:** 1871-10

**Authors:** 


### INDEX OF SUBJECTS.

### ORIGINAL COMMUNICATIONS.

The Hypophosphate in Cholera Infantnm....517
Coma....520
Cephalanthus Occidentalis (Kephalahead Anthos Flower) Globe Flower....523
Chlorate of Pottassa, etc....526
Treatment of Neuralgia....528
Lowndes County (Miss.) Medical Association....530

### EXTRACTS AND GLEANINGS.

Snake Bite Cured by the Application of a Coal of Fire, 532; Sulphite of Soda in Dyspepsia, 533; Tinea Capitis—Bromide of Potassium in "Summer Complaints of Children," 534; Chronic Hydrocephalus—Oil of Horse Chesnuts, 535; Phosphorus in Locomotor Ataxia—Burns and their Treatment, 536; Essence of Turpentine in the Treatment of Wounds—Cholera Infantum, 537; Treatment of Enlarged Tonsils—Gonorrhœa, 538; Local Anæsthesia—Pepsin in the Vomiting of Pregnancy, 539; Iodoform in some Phases of Syphilis—Electricity in Tic Doloreuax and Hemicrania, 540; New Treatment for Small-pox—Valuable Combination—For Morning Sickness, 541; Treatment of Eczema—Antizimotic Treatment of Syphilis, 542; Bromide of Potassium and Fluid Extract Gelseminum in Headache—Raw Beef in the Vomiting of Pregnancy—Mammary Abscess, 543; Tansy in Epistaxis—Treatment of Hydrocele, etc., 544; Iodo-Bromide of Calcicum Compound—A "Specific for Asthma, 545; Treatment of Chilblains—Dysmenorrhœa—Wash for Sore Nipples, 546; Flatulence, etc.—Spermatorrhœa, 547; Sulphate of Berbeeria in Chronic Inflammation of the Uterus—New Remedy for Dysmenorrhœa and Tenesmus of Dysentery, 548; Carbolic Acid in Children's Diseases 549; Iodized Milk—Aconite and Muriate of Ammonia in Neuralgia—Iodine Gargle, 552; New Salve for Chapped Nipples, 553; Anæsthetics—Treatment of Sunstroke, 554; New Plan of Dressing Wounds—Carbolate of Lime in Pertussis, 555; Bismuth in Dysentery, etc., 556; New Use of Potassium—Punciure of the Intestines, etc., 557; Iodoform in Neuralgia, 558; Lycoperdon of Buff Ball as a Styptic and Anæsthetic, 559; Burns and Scalds, 560; Cod Liver Oil in Eczema, 561; Quinia in Hay Asthma, 562; Formulæ for Dyspepsia, 563; Phytolacca Decandra in Cancer—Pomatum for Chapped Lips, 564.

### EDITORIALS, CORRESPONDENCE AND MISCELLANEOUS.

Our Editorial Staff—Our Platform....565
A Quarrel Ended....567
Go to Work Friends....572
The Truth Plainly, Forcibly Written....574
To Our Subscribers....576

